# Discrete Roles for Impulsivity and Compulsivity in Gambling Disorder

**DOI:** 10.3389/fpsyt.2021.789940

**Published:** 2021-12-07

**Authors:** Gemma Mestre-Bach, Trevor Steward, Iris M. Balodis, Elise E. DeVito, Sarah W. Yip, Tony P. George, Brady A. Reynolds, Roser Granero, Fernando Fernandez-Aranda, Susana Jimenez-Murcia, Marc N. Potenza

**Affiliations:** ^1^Health Sciences School, Universidad Internacional de La Rioja, La Rioja, Spain; ^2^Melbourne School of Psychological Sciences, University of Melbourne, Parkville, VIC, Australia; ^3^Department of Psychiatry and Behavioural Neurosciences, McMaster University, Hamilton, ON, Canada; ^4^Department of Psychiatry, Yale University School of Medicine, New Haven, CT, United States; ^5^Yale Child Study Center, Yale University School of Medicine, New Haven, CT, United States; ^6^Addictions Division, Centre for Addiction and Mental Health, University of Toronto, Toronto, ON, Canada; ^7^Division of Brain and Therapeutics, Department of Psychiatry, University of Toronto, Toronto, ON, Canada; ^8^Department of Behavioral Science, University of Kentucky, Lexington, KY, United States; ^9^Ciber Fisiopatología Obesidad y Nutrición (CIBERObn), Instituto de Salud Carlos III, Barcelona, Spain; ^10^Departament de Psicobiologia i Metodologia de les Ciències de la Salut, Universitat Autònoma de Barcelona, Barcelona, Spain; ^11^Department of Psychiatry, Bellvitge University Hospital-IDIBELL, Barcelona, Spain; ^12^Department of Clinical Sciences, School of Medicine and Health Sciences, University of Barcelona, Barcelona, Spain; ^13^Department of Neuroscience, Yale University School of Medicine, New Haven, CT, United States; ^14^The National Center on Addiction and Substance Abuse, Yale University School of Medicine, New Haven, CT, United States; ^15^Connecticut Mental Health Center, New Haven, CT, United States

**Keywords:** gambling disorder, impulsive behaviors, compulsive behaviors, addictive behaviors, delay discounting, set-shifting

## Abstract

**Background and Objective:** Complex associations between gambling disorder (GD) and impulsivity have been identified. However, little is known regarding how compulsivity associates with different impulsivity domains in GD. In this study, we examined associations between self-reported and behavioral measures of impulsivity–assessed through the Barratt Impulsiveness Scale (BIS-11) and the Experiential Discounting Task (EDT), respectively- and compulsivity-measured using the Padua Inventory and the Wisconsin Card Sorting Test (WCST), respectively-, in an adult sample with GD (*N* = 132, 94 men and 38 women, ages ranging from 18 to 69 years). GD severity was assessed using the South Oaks Gambling Screen.

**Methods:** Structural Equation Modeling was used to examine relationships between impulsivity and compulsivity measures, age, and GD severity.

**Results:** BIS-11 non-planning and BIS-11 total scores positively correlated with GD severity. The standardized coefficients for the SEM showed direct positive contributions of BIS-11 non-planning, Padua and EDT scores to GD severity. Only participants' ages directly contributed to WCST perseverative errors, and no direct or indirect effects were found with respect to GD severity.

**Conclusion:** The findings suggest that specific aspects of impulsivity and compulsivity contribute to GD severity. Interventions specifically targeting domains that are most relevant to GD severity may improve treatment outcomes.

## Introduction

Transdiagnostically, impulsivity and compulsivity contribute to the development, maintenance and severity of mental disorders, including gambling disorder (GD) (1–5). Even though impulsivity and compulsivity are distinct, multifaceted constructs, both may involve impaired tendencies to inhibit or delay behaviors and may be present concurrently or at different times in the same disorder (6). While impulsivity and compulsivity had been hypothesized to lie at different ends of a continuous spectrum (7), data suggest that the constructs may be more orthogonal, with elevated levels of each in disorders such as GD (8).

Impulsivity has been defined as a “predisposition toward rapid, unplanned reactions to internal or external stimuli with diminished regard to the negative consequences of these reactions to the impulsive individual or to others” (9, 10). As suggested by this definition, impulsivity is a complex entity and may include components related to pre-potent motor disinhibition [impulsive action (11)] and difficulties in delaying gratification [impulsive choice (12)], and each may relate to specific neurocognitive mechanisms (2, 6, 13). Impulsivity has been implicated in multiple psychiatric disorders and conditions, such as attention-deficit/hyperactivity disorder (14, 15), eating disorders (16–20), obesity (21–23), and substance-use disorders and behavioral addictions (24–26). In GD, high levels of both impulsive action (27) and impulsive choice (26, 28) have been observed, using self-report or behavioral measures, although there is a lack of consistency in explaining their association with GD severity (27, 29–31). GD has also been linked to impulsive tendencies (32, 33). Specifically, positive and negative urgency levels and lack of perseverance are dimensions that may best distinguish individuals with and without GD (34–36).

Compulsivity has been defined as involving, “the performance of repetitive and functionally impairing overt or covert behavior without adaptive function, performed in a habitual or stereotyped fashion, either according to rigid rules or as a means of avoiding perceived negative consequences” (6). Compulsivity has been implicated in multiple mental disorders including obsessive-compulsive disorder (37), trichotillomania (38), and anorexia nervosa (39). In GD, compulsive features have been linked to genetic factors (40), and compulsivity-related impairments in cognitive flexibility may involve difficulty in learning from mistakes and implementing alternative problem-solving methods (41–44). During performance of attentional set-shifting tasks like the Wisconsin card sorting test (WCST) (45), worse performance (less flexibility or more compulsivity) has been observed in individuals with GD vs. those without, as reflected in more perseverative errors (46). Moreover, self-reported compulsivity has been positively associated with GD severity, linked to poor control over gambling-related thoughts and behaviors, and associated with poorer treatment outcomes (47, 48).

Changes in decision-making processes and impulsivity dimensions are affected by neurodevelopment across the lifespan (49–51). More specifically, it has been postulated that greater maturation of mesolimbic circuitry and cognitive control systems occur with development from childhood/adolescence through younger/middle adulthood, thereby reducing the degree to which delayed rewards are devalued (49). In the case of GD, research has found age and GD severity to serve as the best predictors of individual differences in choice impulsivity (26, 52, 53). Regarding compulsivity, differences have been found between different age groups in features such as cognitive flexibility and planning, suggesting maturational and developmental impacts as well as possible effects of cognitive aging in older samples (54, 55).

Therefore, although associations between impulsivity, compulsivity, age, and GD severity have been described, further study of how impulsivity and compulsivity may relate to clinical characteristics of GD is needed. Moreover, in GD, the simultaneous examination of both self-reported and behavioral aspects of both impulsivity and compulsivity has been scarce. Elevated impulsivity and compulsivity have been observed in both self-report and behavioral measures of impulsivity and compulsivity and have been, at times, linked to treatment outcomes (47, 48, 56–60). Although complex relationships between impulsivity and compulsivity have been proposed (8, 56), few studies have concurrently investigated self-reported and behavioral measures of both impulsivity and compulsivity in GD. Finally, little research has examined potential mediating roles of these domains in relationships between age and GD severity.

Here, we examined the interplay between self-reported and behavioral measures of impulsivity and compulsivity and GD severity in adults with GD and used structural equation modeling (SEM) to explore associations between age and these factors. We hypothesized that GD severity levels would positively relate to both self-reported and behavioral measures of impulsivity and compulsivity. We also hypothesized that age would be positively associated with compulsivity, as suggested by previous studies (54, 55), and that impulsivity would show a direct positive association with GD severity, as previously observed (58). Finally, given these relationships, we hypothesized that age would impact impulsivity and compulsivity levels that would then impact GD severity; in other words, impulsivity and compulsivity would mediate relationships between age and GD severity.

## Materials and Methods

### Participants and Procedure

The sample was comprised of 132 participants who met criteria for GD. They were recruited at a University in the Problem Gambling Clinic through advertisements. Individuals 18 years or older with a diagnosis of DSM-IV pathological gambling as determined by structured clinical interview (Structured Clinical Interview for Pathological Gambling) were included (61). The sample included all consecutive subjects who met criteria for GD during the recruitment period (October, 2006 to November, 2015).

### Measures

#### Clinical Characteristics

##### South Oaks Gambling Screen (SOGS)

This questionnaire (62) includes 20 items assessing the frequency, presence and severity of gambling-related activities (scores range from 0 to 20). This questionnaire discriminates between probable non-problem gambling (from 0 to 2), probable problem gambling (from 3 to 4), and probable pathological gambling (from 5 to 20), with higher scores being indicative of greater problem-gambling severity. The SOGS is a widely used instrument to screen for gambling problems in research and clinical settings, and has been used as a measure of GD severity (63). Internal consistency obtained in the study was Cronbach's alpha α = 0.742.

#### Impulsivity

##### The Barratt Impulsiveness Scale (BIS-11)

The BIS-11 (64) is a 30-item, self-report instrument that includes three subscales: (1) attentional, (2) motor, and (3) non-planning. Item responses range from 1 to 4 (Rarely/Never, Occasionally, Often, Almost Always/Always). It has demonstrated adequate test-retest reliability (Spearman's ρ = 0.83) and acceptable internal consistency (α = 0.83), with a score of 72 or higher representing high impulsivity (64). Internal consistency obtained in the study was Cronbach's α = 0.736.

##### Experiential Discounting Task (EDT)

The EDT is a computerized task to assess choice impulsivity, in which subjects experience chose smaller, sooner and certain rewards vs. larger, later, and probabilistic rewards in real time (65). Subjects completed four session blocks associated with different time delays, three of which involved choices between an adjusting and certain amount (initially, $0.15) that was delivered immediately or a standard amount ($0.30) that was delayed and probabilistic (35%). For the other session, there was no delay (0 s), and the reward ($0.30; probability, 35%) was delivered immediately. Choice options were indicated by the “illumination” of light bulbs on the screen. The immediate amount (right side of screen) was adjusted in value in that the amount increased by a set percentage following a delayed standard choice but decreased following an immediate choice. The delayed standard amount (left side of screen) was not adjustable. The standard option choice resulted in a wait of a specified delay (0, 7, 14, and 28 s). If the money was delivered, it could be transferred to the “bank” by clicking on the “illuminated” bank image, which resulted in coin delivery from a coin dispenser. Therefore, participants received real time feedback based on their decisions. For each choice block, subjects made choices until an indifference point was reached, defined as choosing each option (i.e., immediate and delayed) three times within six consecutive choice trials—thus keeping the adjusting amount constant over those six choices. After an indifference point was established or the delayed option was chosen 15 times (reflecting minimal discounting), the session ended. The remaining sessions (i.e., 7, 14, and 28 s) were completed in ascending order.

The plots of the indifference curves (normalized indifference point plotted for each delay interval) for each individual were fit with either an exponential (VS = VAe – kd) or a hyperbolic (VS = VA/1 + kd) function where the subjective value (VS) was a modification of the actual value (VA) by the delay (d) and a discount constant (*k*). The *k*-value represents the steepness of the delay-discounting curve and was used as a measure of choice impulsivity. A higher *k* represents higher choice impulsivity. Curve-fitting was conducted using Prism 5 (GraphPad software). We assessed the proportion of choices for each delay interval [delayed choice ratio = delayed choice/total choice] and compared higher-impulsivity and lower-impulsivity subjects (dichotomized by median *k*).

#### Compulsivity

##### The Padua Inventory

The Padua Inventory (66, 67) is a 60-question self-report instrument that assesses presence and severity of obsessive and compulsive symptomatology. The inventory contains four factors: impaired control over mental activities, which assesses ruminations and exaggerated doubts; fear of contamination; checking; and impaired control over motor activities which measures urges and worries of losing control over motor behaviors. The Padua Inventory has shown high test-retest reliability, high internal consistency, and good convergence validity with other instruments assessing obsessive and compulsive symptomatology (66, 67). Internal consistency obtained in the study was Cronbach's α = 0.967.

##### The Wisconsin Card Sorting Test (WCST)

The Wisconsin Card Sorting Test (WCST) (45) is a set-shifting task designed to assess cognitive flexibility. We used the WCST and scored performance as described previously (68, 69). The WCST assesses tendencies to shift cognitive strategies in response to altering conditions, and in so doing, assesses strategic planning, organized searching and the use of environmental feedback to modify cognitive approaches. The test consists of 128 cards that vary according to three attributes: the number, color, and shape of their elements. Participants are instructed to sort the cards in piles beneath four reference cards that vary in these same dimensions. The only feedback given to the participant is the word “right” or “wrong” after each sorting. Initially, color is the correct sorting category, and positive feedback is given only if the card is placed in the pile with the same color. After 10 sequential correct answers the categorization criteria change. Thus, only classifications that match the new category will result in positive feedback. Participants must learn to change the sorting categories according to the feedback they receive. The test ends after all cards are sorted, or after six full categories are completed. The number of trials completed, the percentage of perseverative errors (i.e., failures to change sorting strategy after negative feedback) and the percentage of non-perseverative errors are recorded.

### Statistical Analysis

Statistical analysis was conducted with Stata17 for Windows (70). First, associations between impulsivity and compulsivity measures and GD severity (SOGS total score) were estimated through bivariate Pearson correlation coefficients (*r*). Due to strong associations between *r*-coefficients and sample size in determining statistical significance, |r| > 0.10–0.24 was considered a low effect size, |r| > 0.24–0.37 was considered a moderate effect size and |r| > 0.37 was considered a large effect size (71).

Second, the associations between impulsivity-compulsivity measures and GD severity were evaluated through path analysis, a straightforward extension of multiple regression modeling used with the aim to estimate the magnitude and significance of hypothesized associations into a set of variables, including mediational links (direct and indirect effects) (72). Path analysis in this study was used as a case of structural equation modeling (SEM), with the maximum-likelihood estimation method of parameter estimation and evaluating goodness-of-fit through standard statistical measures [including the root mean square error of approximation (RMSEA), Bentler's Comparative Fit Index (CFI), the Tucker-Lewis Index (TLI), and the standardized root mean square residual (SRMR)] (73). Adequate model fit was considered non-significant by chi-square (χ^2^) tests and if the following criteria were met (73): RMSEA <0.08, TLI > 0.9, CFI > 0.9, and SRMR <0.1. The global predictive capacity of the model was measured by the coefficient of determination (CD). The study model included impulsivity-compulsivity measures and age as endogenous variables and SOGS total score (i.e., GD severity) as the exogenous variable. Due to the large set of observed variables, with the aim to achieve adequate fitting and a parsimonious model, a pre-selection of the best measures for the impulsivity-compulsivity constructs was done through stepwise multiple regression (the SOGS total score was defined as the criterion and the remaining clinical variables as potential predictors). In addition, a multi-group SEM was tested in the study, including gender as a group variable with the aim to assess the invariance of the structural coefficients.

## Results

### Sample Description

The frequency distribution of the sociodemographic and clinical variables of the study are included in Table 1. Most participants were male (71.2%), White (60.6%), and single (58.3%). Age range in the study was 18–69 years-old, and SOGS total score was between 5 and 20.

**Table 1:** Sample description (*N* = 132).

**Sociodemographics**		***n***	***Perc*.**	
Gender	Female	38	28.8%	
	Male	94	71.2%	
Race		White	60.6%	
		Black	36.4%	
		Other	3.0%	
Marital status	Single	77	58.3%	
	Married	22	16.7%	
	Separated-divorced	33	25.0%	
Education level	Postgraduate	6	4.5%	
	College graduate	25	18.9%	
	Some college without diploma	52	39.4%	
	High school diploma/GED	45	34.1%	
	Less than High School	4	3.0%	
**Chronological age**	**Min**	***Max***	***Mean***	***SD***
Age (years);	18	69	42.77	12.25
Clinical measures	Min	Max	Mean	SD
GD: SOGS total score	5	20	12.10	4.03
WCST trials completed	68	128	102.06	22.04
WCST % perseverative errors	4	44	12.91	8.19
WCST % non-perseverative errors	3	47	14.54	8.85
Padua impaired-control over mental activities	0	44	8.62	10.52
Padua fear of contamination	0	39	9.07	8.94
Padua checking	0	24	5.15	6.25
Padua impaired-control over motor activities	0	17	1.60	3.29
Padua total score	0	107	24.44	24.37
BIS-11 attentional	8	31	15.53	4.08
BIS-11 motor	13	39	24.46	5.19
BIS-11 non-planning	14	40	26.21	5.08
BIS-11 total	38	102	66.20	11.91
EDT k-delay discounting^a^	−0.03	2.50	0.027	0.28
EDT AUC index^a^	0.11	0.47	0.195	0.04

a *Median and SD are reported for this measure*.

*Min, minimum; Max, maximum; SD, standard deviation; GD, gambling disorder; SOGS, South Oaks Gambling Screen; WCST, Wisconsin Card Sorting Test; BIS-11, Barratt Impulsiveness Scale; EDT, Experiential discounting task*.

### Correlations Between Variables

Table 2 contains the correlation matrix with coefficients between the study variables. Associations with effect sizes in the moderate to high range are marked in bold. GD severity positively correlated with BIS-11 non-planning and BIS-11 total scores. EDT-k values were also positively correlated with BIS-11 scores, except for non-planning (the EDT-AUC index was negatively correlated with the EDT-k values). Age was positively related with WCST measures (older age was associated with worse cognitive performance). All remaining significant associations were between subscales of the same questionnaires.

**Table 2:** Correlations between the variables of the study.

		**2**	**3**	**4**	**5**	**6**	**7**	**8**	**9**	**10**	**11**	**12**	**13**	**14**	**15**	**16**
1	SOGS total score	0.08	−0.01	0.04	−0.01	−0.03	−0.07	−0.02	−0.04	0.13	0.21	**0.24^†^**	**0.26^†^**	0.06	0.07	0.14
2	WCST trials completed		**0.72^†^**	**0.78^†^**	0.10	0.03	0.08	−0.03	0.07	0.00	−0.14	0.02	−0.05	0.04	−0.05	**0.36^†^**
3	WCST perseverative errors			**0.66^†^**	0.16	0.10	0.13	0.03	0.14	−0.07	−0.18	−0.03	−0.12	0.02	−0.08	**0.33^†^**
4	WCST non-perseverative errors				0.17	0.03	0.15	0.04	0.13	−0.01	−0.11	0.02	−0.04	0.10	−0.12	**0.32^†^**
5	Padua impaired-control over mental activities					**0.54^†^**	**0.80^†^**	**0.66^†^**	**0.92^†^**	0.21	0.01	0.01	0.08	−0.02	−0.13	−0.04
6	Padua fear of contamination						**0.57^†^**	**0.31^†^**	**0.79^†^**	0.04	−0.10	−0.19	−0.11	0.01	−0.12	−0.06
7	Padua checking							**0.47^†^**	**0.87^†^**	0.09	0.05	−0.13	−0.01	0.04	−0.18	−0.10
8	Padua impaired-control over motor activities								**0.65^†^**	0.20	0.04	−0.02	0.08	0.02	−0.04	−0.14
9	Padua total score									0.16	−0.01	−0.11	0.00	0.01	−0.15	−0.08
10	BIS attentional										**0.56^†^**	**0.48^†^**	**0.79^†^**	**0.25^†^**	−0.06	−0.12
11	BIS motor											**0.55^†^**	**0.86^†^**	**0.28^†^**	−0.07	−0.11
12	BIS non-planning												**0.83^†^**	0.16	−0.07	0.01
13	BIS total													**0.27^†^**	−0.08	−0.08
14	EDT k-delay discounting														**−0.58^†^**	−0.08
15	EDT AUC index															0.01
16	Age (years)															

† *Bold: effect size into the moderate (|R| > 0.24) to high range (|R| > 0.37). Sample size: N = 132. SOGS, South Oaks Gambling Screen; WCST, Wisconsin Card Sorting Test; BIS-11, Barratt Impulsiveness Scale; EDT, Experiential discounting task*.

### Path Analysis

**Figure 1:**
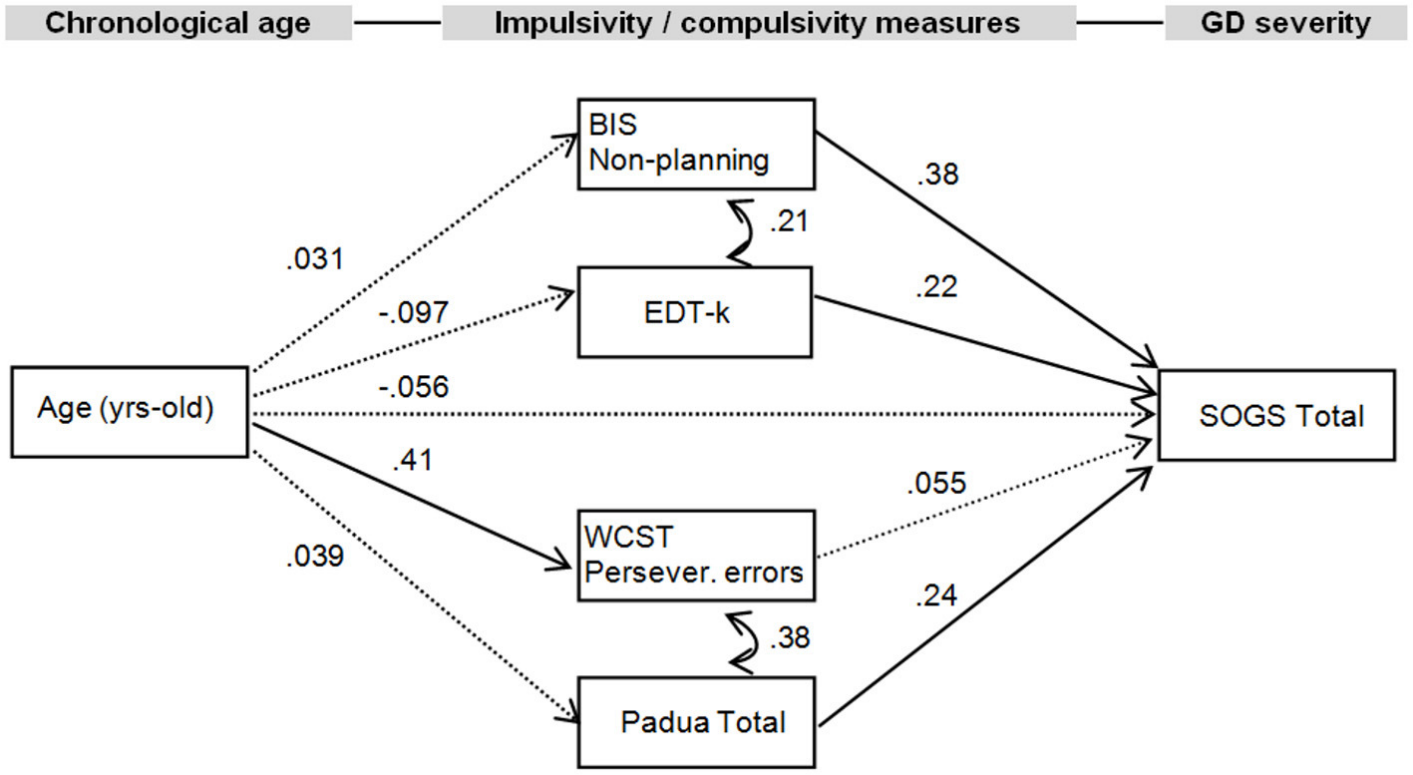
Path diagram with structural equation modeling showing standardized coefficients (*N* = 132). Continuous parameter: significant parameter. Dash line, non-significant parameter; BIS-11, Barratt Impulsiveness Scale; EDT, Experiential discounting task; WCST, Wisconsin Card Sorting Test; SOGS, South Oaks Gambling Screen; Persev., perseverative.

The standardized coefficients for the SEM are included in the diagram in Figure 1, and the complete results of the model testing direct, indirect, and total effects are included in Table 3. The EDT-k values were selected for the EDT task based on the results obtained in the correlation matrix. The joint test measuring the invariance of the structural parameters by gender obtained non-significant results (χ^2^ = 13.02, *p* = 0.162), indicating that the path analysis did no significant difference between men and women (that is, gender did not have a moderating role in the SEM paths). Adequate fitting was obtained for the SEM: χ^2^ = 7.06 (*p* = 0.530), RMSEA = 0.002, CFI = 0.998, TLI = 0.999, and SRMR = 0.053. Global predictive capacity for the model was 18%. The path diagram indicated a direct positive contribution of BIS-11 non-planning, Padua Inventory total, and EDT-k scores to GD severity. Participants' ages only positively contributed to WCST perseverative errors, and no direct or indirect effects were found with respect to GD severity.

**Table 3:** Structural Equation Modeling: direct, indirect and total effects.

			**Direct effects**					**Indirect effects**					**Total effects**				
			***B***	**SE (B)**	***z***	***p***	**St (B)**	***B***	**SE (B)**	***z***	***p***	**St (B)**	***B***	**SE (B)**	***z***	***p***	**St (B)**
PADUA	Age	Female	0.075	0.322	0.23	0.816	0.039	^*^	–			0	0.075	0.322	0.23	0.816	0.039
		Male	−0.160	0.218	−0.74	0.462	−0.079						−0.160	0.218	−0.74	0.462	−0.079
SOGS	PADUA	Female	0.051	0.034	1.51	0.048	0.240	^*^	–			0	0.051	0.034	1.51	0.048	0.240
		Male	0.060	0.016	−0.62	0.047	0.234						0.060	0.016	−0.62	0.047	0.234
	BIS-11-non plan	Female	0.305	0.123	2.47	0.014	0.376	^*^	–			0	0.305	0.123	2.47	0.014	0.376
		Male	0.112	0.086	1.3	0.045	0.134						0.112	0.086	1.3	0.045	0.134
	EDT-k	Female	3.890	2.502	1.55	0.040	0.223	^*^	–			0	3.890	2.502	1.55	0.040	0.223
		Male	2.139	2.188	−0.98	0.038	0.101						2.139	2.188	−0.98	0.038	0.101
	WCST-persev.	Female	0.031	0.098	0.32	0.750	0.055	^*^	–			0	0.031	0.098	0.32	0.750	0.055
		Male	−0.069	0.051	−1.37	0.172	−0.150						−0.069	0.051	−1.37	0.172	−0.150
	Age	Female	−0.023	0.064	−0.36	0.721	−0.056	0.009	0.047	0.19	0.848	0.022	−0.014	0.068	−0.2	0.839	−0.034
		Male	0.070	0.034	2.07	0.039	0.227	−0.012	0.014	−0.87	0.386	−0.040	0.057	0.032	1.77	0.077	0.186
BIS-11-non plan	Age	Female	0.016	0.084	0.19	0.853	0.031	^*^	–			0	0.016	0.084	0.19	0.853	0.031
		Male	−0.024	0.039	−0.61	0.539	−0.066						−0.024	0.039	−0.61	0.539	−0.066
EDT-k	Age	Female	−0.002	0.004	−0.59	0.558	−0.097	^*^	–			0	−0.002	0.004	−0.59	0.558	−0.097
		Male	−0.002	0.002	−1.05	0.293	−0.112						−0.002	0.002	−1.05	0.293	−0.112
WCST-persev.	Age	Female	0.298	0.110	2.7	0.007	0.410	^*^	–			0	0.298	0.110	2.7	0.007	0.410
		Male	0.211	0.067	3.13	0.002	0.318						0.211	0.067	3.13	0.002	0.318

*B, coefficient. SE, standard error; St (B), standardized coefficient*;

* *Parameter estimates constrained to be equal across groups. Sample size: N = 132*.

*SOGS, South Oaks Gambling Screen; BIS-11, Barratt Impulsiveness Scale; EDT, Experiential discounting task; WCST, Wisconsin Card Sorting Test; persev., perseverative; non-plan, non-planning*.

## Discussion

The first aim of the present study was to examine associations between impulsivity, compulsivity, and GD severity in adults with GD. The second goal was to explore the mediating roles of impulsivity and compulsivity levels between age and GD severity by means of a path analysis. GD severity was positively correlated with self-reported impulsivity (BIS-11 non-planning and BIS-11 total scores). The standardized coefficients for the SEM showed a direct positive contribution of self-reported impulsivity (BIS-11 non-planning), behavioral impulsivity (EDT scores) and self-reported compulsivity (Padua total scores) to GD severity. Participants' age only significantly contributed to behavioral compulsivity (WCST perseverative errors), and no effects were found with respect to GD severity.

Regarding impulsivity, behavioral choice impulsivity (assessed using EDT-k) correlated with self-reported impulsivity (assessed using the BIS-11 and correlating with BIS-11 attentional and motor impulsivity subscales, and total score). Previous studies have found weak or no relationships between most facets of motor and choice impulsivity (74, 75). This may partly be explained by the discrepancies between behavioral and self-report measures of impulsivity-related assessments (76), questioning whether these different tools assess the facets of impulsivity they are intended to measure (77, 78). Alternatively, as prior studies have not examined groups with GD, it is possible that these forms of impulsivity are more closely related in individuals with GD than in the general population.

The present findings suggest that self-reported and behavioral measures of compulsivity are not highly correlated. Many instruments assessing compulsivity are based on conceptualizations of obsessive-compulsive disorder (OCD) and may not be ideal for considering compulsivity as a transdiagnostic construct (79, 80), due to, among other things, the clinical and neurobiological differences between GD and OCD (81). However, like impulsivity, compulsivity is likely a multifaceted construct that includes several conceptually and empirically separable features, such as attentional bias/disengagement or failures in contingency-related cognitive flexibility during habit learning (2, 6). As such, each assessment could be measuring different features that may link to clinical characteristics in unique fashions. Results of the partial correlation matrix and the SEM showed that none of the impulsivity dimensions were associated with compulsivity measures in the present study. This finding supports the notion that both are separate constructs, as suggested by previous data (82).

The present study also assessed associations between impulsivity, compulsivity, and GD severity. The SEM showed a direct positive contribution of impulsivity (BIS non-planning and EDT-k) to GD severity. While prior studies have found no correlation between specific dimensions of impulsivity (assessed with the BIS-11) and GD severity (83), others have found that only high attentional and motor impulsivity BIS-11 scores had significant associations with GD severity (84), and others have found, as in the present study, an association between impulsivity and GD severity (58). The seemingly discrepant results may be due to differing characteristics of the samples studied (e.g., sociodemographic or clinical characteristics, cultural contexts) or other factors, and more research is warranted to examine these possibilities.

The SEM also showed a direct positive contribution of compulsivity to GD severity, although only the total score on the Padua Inventory had a significant association with GD severity. Previous studies suggest that performance differences linked to compulsivity may be associated with the development and the maintenance of GD symptomatology. The cognitive inflexibility or the tendency to perseverate on a behavior could, for example, increase the risk for developing GD behavior; alternatively, compulsivity could be a consequence of GD (46). Longitudinal studies are needed to test these possibilities further.

The finding that not all measures of compulsivity showed an association with GD severity coincides with previous studies, which did not find an association between the WCST performance and GD severity (82). These results suggest that impulsivity may contribute more strongly to the acquisition and development of GD than compulsivity, as found in other behavioral addictions (24), although more studies are needed to examine these relationships, especially in a longitudinal fashion.

Finally, sex and age are two sociodemographic factors that should be considered in relationships between impulsivity, compulsivity, and GD severity (26, 51, 85–87). The present study did not observe differences between men and women in the SEM. Age significantly contributed to WCST perseverative errors, consistent with previous findings by identifying a reduction in cognitive flexibility at older ages (54, 55). However, age was not directly associated with any impulsivity measures, as in previous studies, reaffirming that impulsivity is a complex construct and suggesting that more studies focused on compulsivity-related cognitive domains may be needed (51).

### Clinical Implications

These findings have multiple clinical implications. The utility of categorical classifications has been questioned (88), and transdiagnostic features may link more closely to biological constructs (89, 90). For example, impulsivity has been found to link to insular, amygdalar, and hippocampal structures across individuals with GD, those with cocaine-use disorder and those with neither (91). As suggested (9), clinical data focusing on impulsivity and compulsivity may be used to shift toward a more dimensional framework of psychiatric diagnosis and treatment. This approach may lead to improvements in treatment, especially as changes in both impulsivity and compulsivity have been linked to treatment outcomes in GD (47, 48, 92). A dimensional perspective also addresses the critical heterogeneity in the neurobiology of addictions and may help to identify biomarkers suitable for assessment and helpful for advancing personalized medicine approaches (93).

### Limitations and Future Research

The present study has limitations. First, our sample included participants with GD who were not seeking treatment, and this may limit the generalizability of the results to different clinical populations. Future research should include a treatment-seeking sample, as well as a healthy control group, to assess possible differences in these domains between groups. Similarly, examining the validity of these results to other addictions would be another useful contribution for clinicians, as suggested previously (94). Second, the cross-sectional design does not allow for inferences to be made regarding causality or changes in impulsivity and compulsivity over the course of GD. Longitudinal studies are needed to examine these relationships. Future studies focused on impulsivity, compulsivity, and age of onset of GD would be helpful in order to examine whether a switch from impulsivity (in early stages of the addiction course) to compulsivity exists (10, 46). Third, clinical factors of the participants, such as gambling preferences (data on most preferred/problematic form of gambling were not available), comorbidities, and pharmacological treatments, were not taken into account, and they could be associated with performance on both the self-reported instruments and the behavioral tasks used to assess impulsivity and compulsivity. Fourth, a measure of socioeconomic status was not included in sociodemographic measures, although related measures (e.g., education levels) were. Finally, the Padua Inventory originally was designed for clinical populations with OCD. However, it has been linked to clinically relevant aspects of GD in independent samples (35). Nonetheless, a greater focus on new instruments considering compulsivity within a transdiagnostic framework (95) and that are not as focused on OCD may produce findings that could help to clarify relationships with compulsivity in GD populations (79).

## Conclusions

This study provides greater understanding of how impulsivity and compulsivity may relate to GD severity. Our findings suggest impulsivity and compulsivity are multifaceted and separable constructs and not all impulsivity and compulsivity domains relate equally to GD severity. The findings suggest that these two multifactorial constructs deserve greater attention in both research and clinical settings. Interventions specifically targeting domains that are most relevant to the maintenance of GD may help improve treatment outcomes.

## Data Availability Statement

The raw data supporting the conclusions of this article will be made available by the authors, without undue reservation.

## Ethics Statement

The studies involving human participants were reviewed and approved by Yale School of Medicine Human Investigations Committee. The patients/participants provided their written informed consent to participate in this study.

## Author Contributions

GM-B drafted the manuscript. MP oversaw study design and data collection. IB, ED, SY, TG, and BR contributed to study design or data collection. GM-B, TS, RG, FF-A, SJ-M, and MP planned analyses or conducted analyses. All authors approved the manuscript.

## Funding

MP's involvement was supported by a National Center for Responsible Gaming Center of Excellence grant and by the Connecticut Council on Problem Gambling and the Connecticut Department of Mental Health and Addiction Services. GM-B was supported by a FUNCIVA postdoctoral grant. This research was funded by Ministerio de Ciencia, Innovación y Universidades (grant RTI2018-101837-B-100), FIS PI20/132, and FIS PI17/01167, which received aid from the Instituto Salud Carlos III (Ministerio de Sanidad, Servicios Sociales e Igualdad). The research was also funded by the Delegación del Gobierno para el Plan Nacional sobre Drogas (2017I067 and 2019I47), CIBER Fisiología Obesidad y Nutrición (CIBERobn) is an initiative of ISCIII. We thank CERCA Programme/Generalitat de Catalunya for institutional support. Fondo Europeo de Desarrollo Regional (FEDER) “Una manera de hacer Europa”/“A way to build Europe”.

## Conflict of Interest

MP has consulted for Opiant Pharmaceuticals, Idorsia Pharmaceuticals, AXA, and Game Day Data; has been involved in a patent application with Yale University and Novartis. FF-A received consultancy honoraria from Novo Nordisk and editorial honoraria as EIC from Wiley. The remaining authors declare that the research was conducted in the absence of any commercial or financial relationships that could be construed as a potential conflict of interest.

## Publisher's Note

All claims expressed in this article are solely those of the authors and do not necessarily represent those of their affiliated organizations, or those of the publisher, the editors and the reviewers. Any product that may be evaluated in this article, or claim that may be made by its manufacturer, is not guaranteed or endorsed by the publisher.
